# Relative Importance of Heart Failure Events Compared to Stroke and Bleeding in AF Patients

**DOI:** 10.3390/jcm10050923

**Published:** 2021-02-28

**Authors:** Sandro Ninni, Gilles Lemesle, Thibaud Meurice, Olivier Tricot, Nicolas Lamblin, Christophe Bauters

**Affiliations:** 1 Institut Cœur Poumon, University of Lille, CHU Lille, 59000 Lille, France; gilles.lemesle@chru-lille.fr; 2 Hôpital Privé Le Bois, 59003 Lille, France; tmeurice@me.com; 3 Centre Hospitalier de Dunkerque, 59240 Dunkerque, France; oliviertricot@gmail.com; 4 Institut Pasteur, U1167, Inserm, University of Lille, CHU Lille, 59000 Lille, France; nicolas.lamblin@chru-lille.fr (N.L.); christophe.bauters@chru-lille.fr (C.B.)

**Keywords:** atrial fibrillation, heart failure, stroke, bleeding

## Abstract

Introduction: Incident heart failure (HF), ischemic stroke and systemic embolism (IS/SE), and major bleeding related to anticoagulation therapy are still the most frequent events occurring in patients with atrial fibrillation (AF). The aim of this study was to assess the 3-year incidence, predictors, and related mortality of IS/SE, major bleeding, and HF in a large cohort of AF outpatients. Methods and results: We studied 4973 outpatients with prevalent AF included in the CARDIONOR registry. The mean age was 72.9 ± 11.2 years, 24.1% had diabetes mellitus and 78.9% had anticoagulant therapy at baseline. The mean CHA_2_DS_2_Vasc score was 3.4 ± 1.7. After a median follow-up of 3.2 years (IQR: 2.8 to 3.5), incident HF, IS/SE and major bleeding occurred in 10.5%, 3.3% and 2.1% of patients, respectively. When analyzed as time-dependent variables, IS/SE, major bleeding and hospitalization for decompensated HF were all strongly associated with mortality. The independent predictors of incident HF were age, women, hypertension, diabetes mellitus, coronary artery disease and a previous history of HF. A sensitivity analysis in patients without history of HF at inclusion revealed that incident HF remained the most frequent adverse event, occurring in 5.3% of patients, compared to IS/SE (1.7%) and major bleeding (2.5%). Conclusion: HF is a common residual cardiovascular adverse event occurring in AF outpatients and is associated with a very high mortality. Since modifiable risk factors are associated with incident HF, upstream intensive management of these risk factors would be of interest.

## 1. Introduction

Atrial fibrillation (AF) is the most common arrhythmia worldwide, and is associated with an increased risk of stroke and systemic embolism (IS/SE), heart failure (HF), myocardial infarction and subsequent mortality [1,2,3,4]. The current management of AF mainly relies on anticoagulation and AF risk factor management, which has importantly improved patients’ prognosis, especially regarding the risk of IS/SE [2]. However, residual major cardiovascular events still occur overtime in AF patients. These life-threatening events are especially represented by HF, IS/SE and major bleeding related to anticoagulation therapy [5,6,7]. Although most of the studies focused on each event separately in the setting of randomized control trial cohorts, only a few studies have so far assessed the relative incidence of hospitalization for decompensated HF compared to IS/SE and major bleeding in clinical practice [8]. Furthermore, data regarding the mortality associated with each individual event in the setting of real-life AF management are lacking.

Thus, the purpose of this study was to assess the 3-year incidence, predictors, and related mortality of IS/SE, major bleeding, and HF in a large cohort of AF outpatients.

## 2. Methods

### 2.1. Study Population

This study is a post-hoc analysis from the CARDIONOR study. The CARDIONOR study is a multicenter registry that prospectively enrolled 10,517 consecutive outpatients with a diagnosis of coronary artery disease (CAD), AF, and/or HF between January 2013 and May 2015 [9]. The study flow chart is provided in the Supplementary Materials (Figure S1). The patients were included by 81 cardiologists from the French Region of Nord-Pas-de-Calais during outpatient visits. Documented CAD was defined as a history of myocardial infarction (MI), coronary revascularization, and/or the presence of coronary stenosis >50% on a coronary angiogram. Documented AF was defined as a history of AF, even if in sinus rhythm at inclusion. HF was defined as a history of hospitalization for HF and/or a history of symptoms and signs of HF associated with echocardiographic evidence of systolic dysfunction, left ventricular hypertrophy, left atrial enlargement, or diastolic dysfunction [10]. The sole exclusion criterion was age <18 years. Patients with other cardiovascular or non-cardiovascular illnesses or co-morbidities were not excluded.

A case record form was completed at the initial visit with information regarding the demographic and clinical details of the patients, including current medications. The treating cardiologists then followed up with the patients, with the number of outpatient visits at clinician discretion. Protocol-specified follow-up was performed at 3 years using a standardized case record form to report clinical events. In the case of missing information, a research technician contacted general practitioners and/or patients. The identification of patients with events for adjudication was based on interviews with patients/relatives during outpatient visits, discharge summaries for hospitalization during follow-up that were sent to treating cardiologists, and information obtained by the research technician. The events that patients reported were systematically confirmed from the medical reports.

This study was approved by the French medical data protection committee and authorized by the Commission Nationale de l’Informatique et des Libertés for the treatment of personal health data. All patients consented to the study after being informed in writing of the study’s objectives and treatment of the data, as well as about their rights to object and about access and rectification.

### 2.2. Patient and Public Involvement Statement

The patients and public were not involved in the creation of the study design, recruitment, or statistical analysis. Patients were not consulted to develop patient-relevant outcomes or interpret the results. Patients were not invited to contribute to the writing or editing of this document for readability or accuracy.

### 2.3. Event Definitions during Follow-Up

Among the 10,517 outpatients included in the CARDIONOR registry, a total of 4973 had prevalent AF.

IS was defined as a sudden onset of focal neurological symptoms with the presence of cerebral infarction in the appropriate territory on brain imaging (CT or MRI), regardless of the duration of symptoms (less than or more than 24 h) [11,12]. Systemic embolism was defined as acute loss of blood flow to a peripheral artery, confirmed by magnetic resonance imaging or computed tomography.

Hospitalization for HF was defined as hospitalization for symptoms of dyspnea or edema associated with bilateral rales, elevated venous pressure, interstitial or alveolar edema on chest X-ray, or the addition of intravenous diuretics or inotropic medications [9].

Bleeding events were classified using the Bleeding Academic Research Consortium’s (BARC) definitions [13]. BARC type 1 and 2 bleeds were not collected in our registry. For the purpose of this study, we defined major bleeding as all BARC type ≥3 events.

All clinical events were adjudicated by two investigators blinded to each other. A third investigator joined the adjudication in case of disagreement according to the prespecified definitions above. A consensus was then reached.

### 2.4. Statistical Analysis

Continuous variables are described as mean ± standard deviation (SD). Categorical variables are presented as absolute numbers and/or percentages. The incidence of each cardiovascular event was estimated with the cumulative incidence function, with death as the competing event. Univariable and multivariable assessments of baseline variables associated with each cardiovascular event were performed with the use of a cause-specific hazard model. Hazard ratios (HRs) and 95% confidence intervals (CIs) were calculated. The proportional hazards assumption was tested visually using Kaplan–Meier curves and by examining plots of −ln [−ln (survival time)] against the ln (time). For continuous variables, the linearity assumption was assessed by plotting Schoenfeld residuals versus time. Collinearity was excluded by constructing a correlation matrix between candidate predictors. The associations between incident cardiovascular event and mortality were assessed with Cox analyses, and incident cardiovascular events were modeled as a time-dependent variable. HRs and 95% CIs were calculated. All statistical analyses were performed using STATA 14.2 software (STATA Corporation, College Station, TX, USA). Significance was assumed at *p* < 0.05.

## 3. Results

### 3.1. Baseline Characteristics

As shown in Table 1, the mean age of the study population was 72.9 ± 11.2 years with a slight majority of men (56.3%). All patients were included in an outpatient setting. Most patients had permanent AF (38.4%) or paroxysmal AF (35.8%). Most patients had non-valvular AF (89.9%). A history of hypertension was documented in 66.1% of the cases and 24.1% of the patients had diabetes mellitus. The mean left ventricular ejection fraction (LVEF) was 58 ± 11%; 16.8% of the patients had LVEF < 50% and 33.6% of the patients had a history of HF. Significant CAD was documented in 25.8% of the cases at inclusion; 8.5% of the patients had a previous stroke and 12.4% had a history of peripheral artery disease (PAD). The mean CHA_2_DS_2_-VASc score was 3.4 ± 1.7. Medications at baseline are shown in Table 1. Most patients received at least one antithrombotic treatment (92.6%). Oral anticoagulation (OAC) was used in 78.9% (vitamin K antagonists (VKA), 57.3%; direct oral anticoagulant (DOAC), 21.6%).

### 3.2. Incident Events

**Figure 1:**
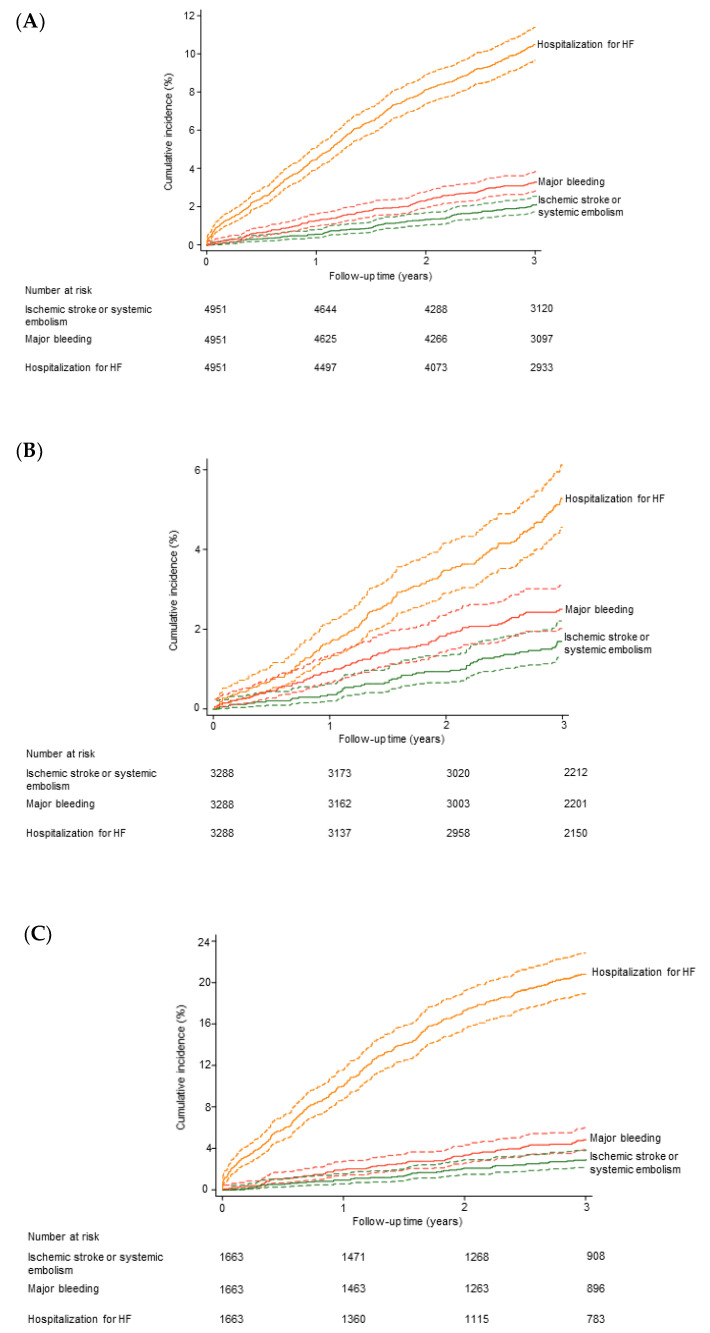
Incident events during follow-up. Cumulative incidence functions (death as the competing event) are shown. (**A**) Overall study population (*n* = 4951 with follow-up). (**B**) Patients without HF at inclusion (*n* = 3288 with follow-up). (**C**) Patients with HF at inclusion (*n* = 1663 with follow-up). HF, heart failure.

A clinical follow-up was obtained at a median of 3.2 (interquartile range: 2.8 to 3.5) years in 4951 (99%) of the 4973 AF outpatients. During the follow-up period, there were 979 deaths among the 4973 patients. The 3-year cumulative risk of death was 17.5% (95% CI: 16.5 to 18.7). The numbers of patients with IS/SE, major bleeding and hospitalization for decompensated HF during follow-up were 120, 179 and 565, respectively. Among the 801 patients who presented at least one incident event, 62 presented at least two different events. The overlap between incident events is provided in the Supplementary Materials (Figure S2). Cumulative incidences including death as the competing event are shown in Figure 1A. The three-year cumulative incidences were 2.1% (95% CI: 1.7 to 2.5), 3.3% (95% CI: 2.8 to 3.8) and 10.5% (95% CI: 9.7 to 11.4), for IS/SE, major bleeding and hospitalization for decompensated HF, respectively.

### 3.3. Correlates of Incident Events

The correlates of IS/SE, major bleeding, and hospitalization for decompensated HF are shown in the Supplementary Materials (Table S1). Four clinical variables were independently associated with IS/SE: older age, history of HF, previous stroke, and history of PAD. Five variables were independently associated with major bleeding: older age, diabetes mellitus, valvular AF, history of HF, and previous stroke. Seven variables were independently associated with hospitalization for decompensated heart failure: older age, women, hypertension, diabetes mellitus, lower LVEF, history of CAD, and history of HF (Table 2).

### 3.4. Outcome after Incident Events

At the end of the follow-up period, 56 patients out of the 120 with incident IS/SE (46.7%), 91 patients out of the 179 with incident major bleeding (50.8%), and 241 patients out of the 565 with incident hospitalization for decompensated HF (42.7%), had died.

**Figure 2:**
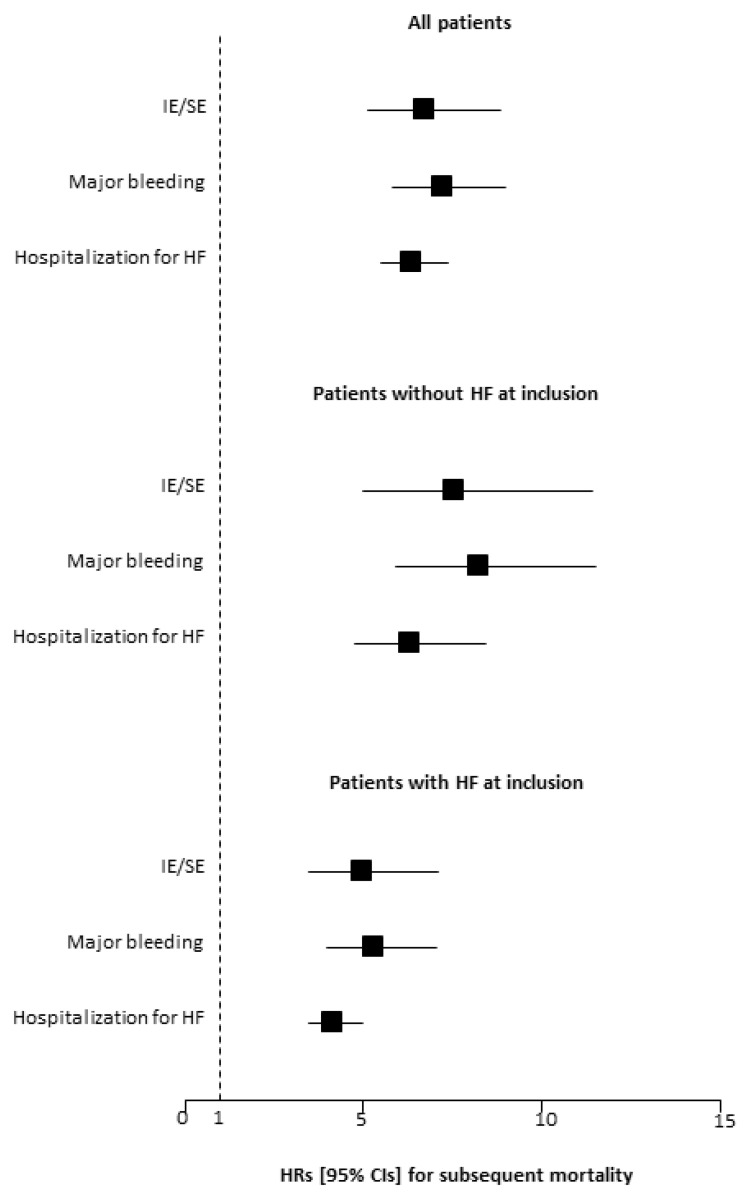
Impact of incident events on subsequent mortality during the follow-up period. Ischemic stroke/systemic embolism (IS/SE), major bleeding, and hospitalization for heart failure (HF) were analyzed as time-dependent variables. HR, hazard ratio; CI, confidence interval.

When analyzed as time-dependent variables, IS/SE, major bleeding and hospitalization for decompensated HF were all strongly associated with mortality. The unadjusted HRs for mortality were 6.72 (95% CI: 5.12 to 8.83; *p <* 0.001) for IS/SE, 7.21 (95% CI: 5.8 to 8.98; *p <* 0.001) for major bleeding, and 6.36 (95% CI: 5.48 to 7.37; *p <* 0.001) for hospitalization for decompensated HF (Figure 2). When adjusted for baseline characteristics (age, gender, hypertension, diabetes mellitus, LVEF, history of CAD, history of HF, previous stroke and history of PAD), the HRs for mortality were 5.19 (95% CI: 3.94 to 6.83; *p <* 0.001) for IS/SE, 5.16 (95% CI: 4.14 to 6.44; *p <* 0.001) for major bleeding, and 3.67 (95% CI: 3.12 to 4.31; *p <* 0.001) for hospitalization for decompensated HF.

### 3.5. Sensitivity Analyses in Patients without History of HF at Inclusion

The analysis was rerun in the 3304 AF patients who had no history of HF at inclusion into the study. There were 420 deaths during follow-up. The 3-year cumulative risk of death was 10.9% (95% CI: 9.9 to 12.1). The numbers of patients with IS/SE, major bleeding and hospitalization for decompensated HF during follow-up were 68, 92 and 188, respectively. Cumulative incidences including death as the competing event are shown in Figure 1B. The three-year cumulative incidences were 1.7% (95% CI: 1.3 to 2.2), 2.5% (95% CI: 2.0 to 3.1) and 5.3% (95% CI: 4.5 to 6.1), for IS/SE, major bleeding, and hospitalization for decompensated HF, respectively. The cumulative incidences including death as the competing event in HF patients are shown in Figure 1C.

The results of time-dependent analyses were consistent with those obtained in the overall study population. The age- and sex-adjusted HR for all-cause mortality were 6.00 (95% CI: 3.96 to 9.10; *p <* 0.001) for IS/SE, 6.77 (95% CI: 4.84 to 9.46; *p <* 0.001) for major bleeding, and 4.60 (95% CI: 3.44 to 6.14; *p <* 0.001) for hospitalization for decompensated HF.

## 4. Discussion

Incident cardiovascular events in AF patients are a great challenge for physicians, since AF itself and the need for anticoagulation therapy often complicate the management of these events. Therefore, cardiovascular event prevention in AF patients is of major interest.

In this large cohort study, we first reported a high incidence of residual cardiovascular events in AF outpatients, with incident hospitalization for decompensated HF representing the most frequent event, reaching 10.5% at 3 years, when compared to major bleeding and IS/SE, with 3-year incidences of 3.3% and 2.1%, respectively.

### 4.1. HF as the Main Residual Event In AF Outpatients

Our results highlight that incident hospitalization for decompensated HF represents a common issue encountered in AF patients. The association between AF and HF has been well described in previous studies. Beyond sharing common predisposing risk factors such as diabetes mellitus, hypertension, ischemic heart disease, and valvular heart disease, AF and HF are closely intertwined, with each disease predisposing to the other [5,14]. Furthermore, when present in combination, AF and HF portend a worse prognosis than either condition alone [14,15]. Importantly, although the association between AF and incident HF has been previously described, many studies did not integrate death as a competing event, which could result in an HF incidence overestimation. Therefore, our statistical design provides a residual risk for incident HF in AF outpatients in clinical practice. Of note, hospitalizations for decompensated HF incidence in AF outpatients is importantly conditioned by the HF status at baseline. Interestingly, our sensitivity analysis provides additional data showing an important risk of incident HF, irrespective of HF status at baseline. This point has been previously observed by Santhanakrishnan et al., who reported, in a Framingham cohort including patients with prevalent AF free of prevalent HF at baseline, a risk for incident HF of 31.4/1000 person-years [14].

This high incidence of HF also emphasizes the prevention of this event as an important unmet need requiring active upstream screening and management in AF patients.

Our results confirm the well-established risk factors for the development of HF, most of which have also been described in relation to AF incidence. Importantly, we found that incident HF was associated with modifiable risk factors, which were hypertension and diabetes.

Cardiovascular risk factor management in AF patients represents an important part of the healthcare plan provided through the Atrial Fibrillation Better Care (ABC) pathway [2]. Furthermore, previous studies have demonstrated the positive effect of the intensive upstream management of modifiable risk factors in the setting of AF prevention or AF ablation [16,17,18]. Although the ABC pathway in its global approach was previously associated with better long-term outcomes [19], the specific impact of risk factors management in preventing HF in AF patients remains unclear.

Incident HF prevention in AF patients was mainly assessed in patients with AF and HF through a rhythm control management strategy and/or AF ablation [20]. However, no specific recommendation to prevent HF in AF patients without a history of HF is currently available. Therefore, the presence of modifiable risk factors associated with incident HF suggests the potential interest in the intensive management of these factors to prevent HF (e.g., SGLT 2 inhibitors prescription in AF diabetic patients).

At last, our study shows that, in addition to being frequent, incident HF is associated with a very high mid-term mortality. Consequently, incident HF in AF patients should be interpreted as a shift in patients evolution, and should encourage physicians to adapt the follow-up for patients at risk.

### 4.2. Stroke and Bleeding Are Less Frequent but Have a Major Impact

Our study also provides contemporary data reflecting clinical practice regarding the residual risk of stroke and bleeding in AF outpatients, with a relatively low incidence of these two events compared to HF.

Of note, these data should be interpreted in the setting of a cohort reflecting clinical practice with an 80% rate of anticoagulant prescription and 30% of patients taking DOAC at the time the study was performed. This proportion of anticoagulated patients is, however, comparable to previous large-scale AF registries, such as the GARFIELD registry, reporting 70% of anticoagulation and a two-thirds use of VKA in anticoagulated patients during the same period, with a continual increase in DOAC use over time [21].

We report an incidence of stroke in line with previous registries. Recently, Maeda et al. reported a residual incidence of stroke of 2% at 3 years in a cohort of 11,848 patients displaying a mean CHA_2_DS_2_Vasc score of 1.4 for males and 2.6 for females [22]. Interestingly, the mean CHA_2_DS_2_Vasc score was 3.4 in our cohort, with a significant proportion of valvular AF representing 10% of patients. Although a higher risk for stroke would be expected because of this higher CHA_2_DS_2_Vasc score in our cohort, the residual stroke risk was similar. This point is also coherent with recent real-life data highlighting a homogenous residual risk for stroke in the setting of a high rate of anticoagulation for patients displaying CHaDsVasc scores below 4 [23].

Our data also reflect the extremely high mortality associated with both ischemic stroke and bleeding. This high mortality and the lack of modifiable risk factors associated with these events should therefore encourage physicians to optimize a possible anticoagulant therapy in AF patients using, preferentially, DOAC.

### 4.3. Study Limitations

Our study has some limitations. First, our data reflect the practice in a regional area, and we do not know whether these findings are generalizable for practices in other parts of the world. Second, because cardiologists determined inclusion, the data may not be generalizable to the overall population with AF in the community. On the other hand, the absence of exclusion criteria, the very high follow-up rate, and the adjudication of clinical events can be considered strengths of the study. Since the CARDIONOR study was primarily designed to assess outcomes in HF, AF and CAD patients, the BARC classification was used to define major bleedings. However, a consistent correlation between severe BARC and ISTH bleeding was previously observed [24], and the use of BARC classification should therefore have a limited impact on the analysis. Furthermore, since all patients were part of the CARDIONOR registry, it is possible that the AF subpopulation was thereby enriched with CAD and/or HF and at a high risk for incident HF.

## 5. Conclusions

HF is the most frequent residual cardiovascular adverse event occurring in AF outpatients (when compared to bleeding and IS/SE), and is associated with a major mortality rate. Its impact in AF patients contemporarily managed with extensive anticoagulation therapy is critical. Since modifiable risk factors are associated with incident HF, the upstream intensive management of these risk factors would be of interest.

## Figures and Tables

**Table 1:** Baseline characteristics of the study population (*n* = 4973).

Age, years	72.9 ± 11.2
Women	2173 (43.7%)
Hypertension	3288 (66.1%)
Diabetes mellitus	1200 (24.1%)
Type of AF:	
- First-diagnosed	293 (5.9%)
- Paroxysmal	1782 (35.8%)
- Persistent	990 (19.9%)
- Permanent	1908 (38.4%)
CHA2DS2Vasc score	3.4 ± 1.7
0/9 or 1/9 gender related	308 (6)
Underlying mechanical prosthetic valve or moderate to severe mitral stenosis	505 (10.1%)
LVEF, %	58 ± 11
History of CAD	1282 (25.8%)
History of HF	1669 (33.6%)
Preserved EF (≥50%)	998 (60%)
Mid-range EF (40 to 49%)	323 (19.4%)
Reduced EF (<40%)	343 (20.6%)
Previous stroke	421 (8.5%)
History of PAD	618 (12.4%)
Previous ICD	171 (3.4%)
Previous CRT	36 (0.7%)
Medications at inclusion:	
- Antiarrhythmic drug	1683 (33.8%)
- Antiplatelet drug	1276 (25.7%)
- Oral anticoagulant	3925 (78.9%)
- Antiplatelet and oral anticoagulant	594 (11.9%)
- Vitamin K antagonist	2850 (57.3%)
- Direct oral anticoagulant	1075 (21.6%)
- Angiotensin-converting enzyme inhibitor or angiotensin receptor blocker	3253 (65.4%)
- Beta-blocker	3469 (69.8%)
- Calcium blocker	1120 (22.5%)
- Digoxin	462 (9.3%)
- Diuretic	2510 (50.5%)
- Aldosterone antagonist	388 (7.8%)
- Statin	2565 (51.6%)
- Insulin	279 (5.6%)
- Oral hypoglycemic drug	871 (17.5%)

Data are mean ± SD or n (percentages). AF, atrial fibrillation; LVEF, left ventricular ejection fraction; CAD, coronary artery disease; CRT: cardiac resynchronization therapy; HF, heart failure; ICD: implantable cardioverter defibrillator; PAD, peripheral artery disease.

**Table 2:** Multivariable analysis for correlates of incident events in outpatients with atrial fibrillation.

	Ischemic Stroke/Systemic Embolism	Major Bleeding	Hospitalization for Decompensated HF
Age (per year)	1.04 [1.02–1.06] *p* < 0.001	1.03 [1.02–1.05] *p* < 0.001	1.04 [1.03–1.05] *p* < 0.001
Women	-	-	1.21 [1.01–1.45] *p* = 0.044
Hypertension	-	-	1.32 [1.08–1.60] *p* = 0.006
Diabetes mellitus	-	1.41 [1.02–1.94] *p* = 0.037	1.60 [1.34–1.91] *p* < 0.001
Permanent AF	-	-	-
Underlying mechanical prosthetic valve or moderate to severe mitral stenosis		1.66 [1.12–2.46] *p* = 0.011	
LVEF (per %)	-	-	0.97 [0.96–0.98] *p* < 0.001
History of CAD	-	-	1.30 [1.08–1.56] *p* = 0.005
History of HF	1.57 [1.09–2.25] *p* = 0.016	1.83 [1.36–2.47] *p* < 0.001	2.83 [2.31–3.47] *p* < 0.001
Previous stroke	2.23 [1.39–3.59] *p* = 0.001	1.65 [1.06–2.55] *p* = 0.026	-
History of PAD	1.84 [1.19–2.84] *p* = 0.006	-	-

Data are hazard ratios [95% confidence intervals] and *p* values. Multivariable analysis was performed using a stepwise approach with forward selection (the *p* value for entering into the model was set at 0.05). HF, heart failure; AF, atrial fibrillation; LVEF, left ventricular ejection fraction; CAD, coronary artery disease; PAD, peripheral artery disease.

## Data Availability

Data are available from the corresponding author on reasonable request.

## Supplementary Materials

The following are available online at https://www.mdpi.com/2077-0383/10/5/923/s1, Figure S1: Study flow chart, Figure S2: Overlap between incident events, Table S1: Univariable analysis for correlates of incident events in outpatients with atrial fibrillation.

## Abbreviations

AF	atrial fibrillation
DOAC	direct oral anticoagulant
HF	heart failure
LVEF	left ventricular ejection fraction
PAD	peripheral artery disease
VKA	vitamin K antagonist
