# Supplementary material for: Relative Importance of Heart Failure Events Compared to Stroke and Bleeding in AF Patients
Source: J Clin Med. 2021 Feb 28;10(5):923. doi: 10.3390/jcm10050923 (PMC7957734; doi:10.3390/jcm10050923)
Supplement: Supplementary file 1 [file jcm-10-00923-s001.pdf]

Supplementary material

Figure S1. Study flow chart

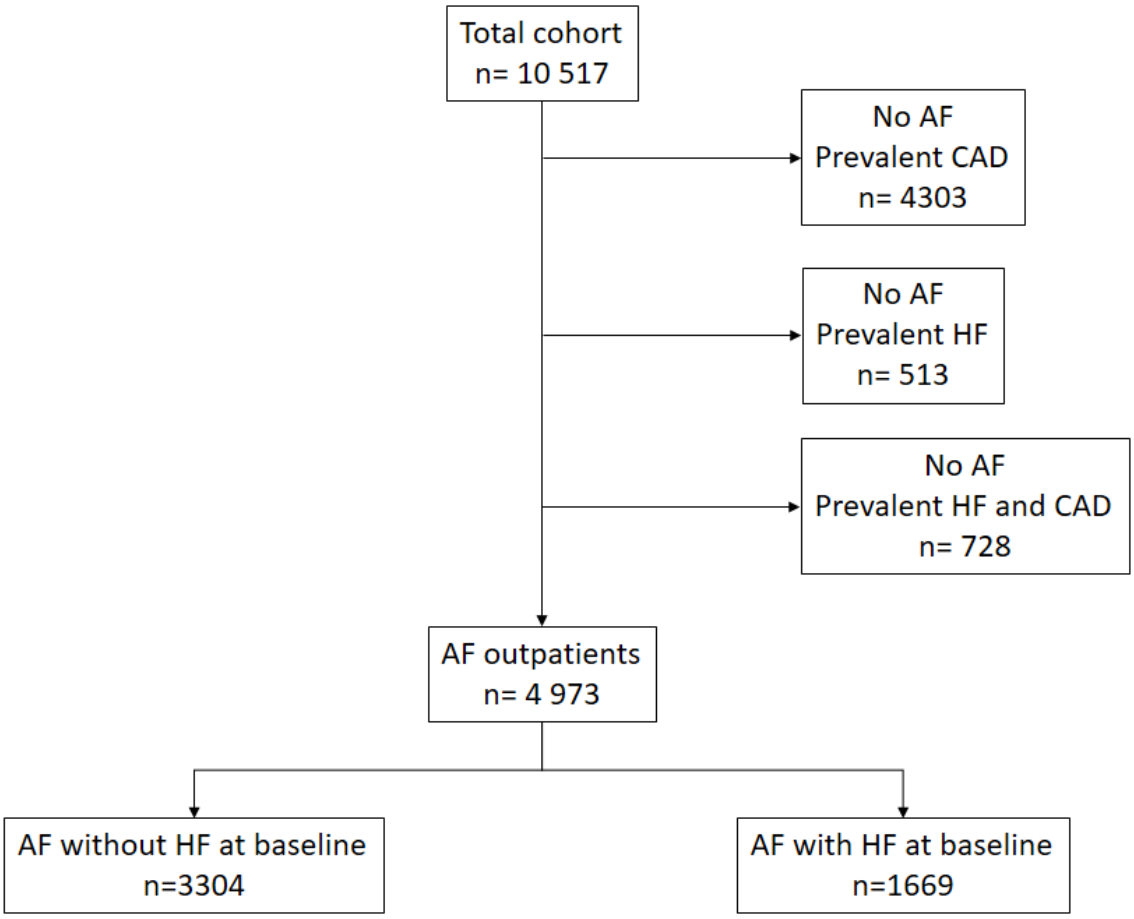

AF: atrial fibrillation, HF: Heart failure, CAD: coronary artery disease

Figure S2. Overlap between incident events

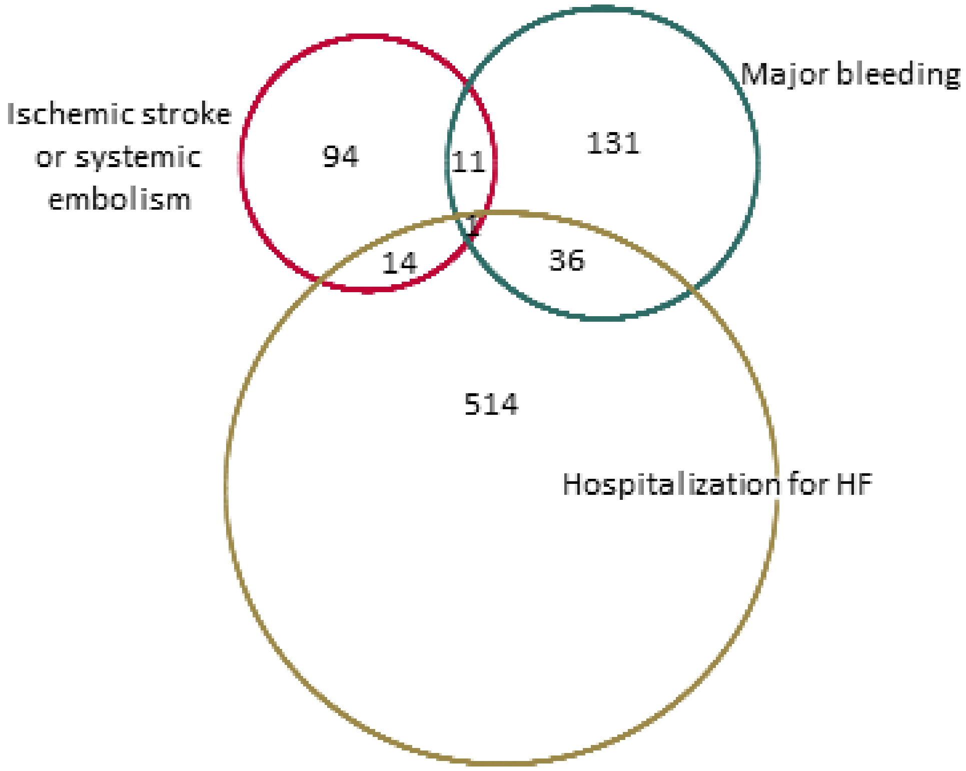

Table S1. Univariable analysis for correlates of incident events in outpatients with atrial fibrillation.

	Ischemic stroke/systemic embolism	Major bleeding	Hospitalization for decompensated HF
	Univariable	Univariable	Univariable
Age (per year)	1.04 [1.02-1.06]<br>*P* < 0.001	1.03 [1.02-1.05]<br>*P* < 0.001	1.04 [1.03-1.05]<br>*P* < 0.001
Women	1.29 [0.90 – 1.85]<br>*P* = 0.163	1.02 [0.76-1.36]<br>*P* = 0.917	1.09 [0.92-1.28]<br>*P* = 0.332
Hypertension	1.02 [0.70-1.49]<br>*P* = 0.920	1.37 [0.99-1.90]<br>*P* = 0.060	1.59 [1.31-1.92]<br>*P* < 0.001
Diabetes mellitus	1.53 [1.04-2.25]<br>*P* = 0.031	1.45 [1.05-1.99]<br>*P* = 0.023	1.87 [1.57-2.22]<br>*P* < 0.001
Permanent AF	1.73 [1.21-2.48]<br>*P* = 0.003	1.66 [1.24-2.22]<br>*P* = 0.001	1.69 [1.44-2.00]<br>*P* < 0.001
Underlying mechanical prosthetic valve or moderate to severe mitral stenosis	1.41 [0.83-2.39]<br>*P* = 0.199	1.92 [1.30-2.82]<br>*P* = 0.001	1.41 [1.11-1.80]<br>*P* = 0.005
LVEF (per %)	0.99 [0.97-1.01]<br>*P* = 0.135	0.98 [0.97-0.99]<br>*P* < 0.001	0.95 [0.94-0.96]<br>*P* < 0.001
History of CAD	1.55 [1.06-2.27]<br>*P* = 0.023	1.51 [1.11-2.06]<br>*P* = 0.009	2.17 [1.83-2.56]<br>*P* < 0.001
History of HF	1.75 [1.22-2.52]<br>*P* = 0.002	2.13 [1.59-2.86]<br>*P* < 0.001	4.86 [4.01-5.79]<br>*P* < 0.001
Previous stroke	2.46 [1.53-3.94]<br>*P* < 0.0001	1.70 [1.10-2.64]<br>*P* = 0.017	1.21 [0.91-1.60]<br>*P* = 0.189
History of PAD	2.11 [1.37-3.26]<br>*P* = 0.001	1.52 [1.03-2.25]<br>*P* = 0.037	1.58 [1.26-1.96]<br>*P* < 0.001
Oral anticoagulation	0.84 [0.55-1.27]<br>*P* = 0.402	1.10 [0.76-1.59]<br>*P* = 0.612	1.50 [1.19-1.88]<br>*P* = 0.001

Data are hazard ratios [95% confidence intervals] and *P* values. Multivariable analysis was performed using a stepwise approach with forward selection (the *P* value for entering into the model was set at 0.05). HF, heart failure; AF, atrial fibrillation; LVEF, left ventricular ejection fraction; CAD, coronary artery disease; PAD, peripheral artery disease.
